# Supplementary material for: Identification of photoperiod-induced specific miRNAs in the adrenal glands of Sunite sheep (Ovis aries)
Source: Front Vet Sci. 2022 Jul 22;9:888207. doi: 10.3389/fvets.2022.888207 (PMC9354845; doi:10.3389/fvets.2022.888207)
Supplement: Supplementary file 1 [file Data_Sheet_1.ZIP › Supplementary file/miRDeep2 file.zip!/miRDeep2 file/Know_Structure/LP42A2/oar-mir-181a-1.pdf]

miRBase precursor : oar-mir-181a-1
Total read count : 21597
oar-miR-181a read count : 21507
remaining reads : 90

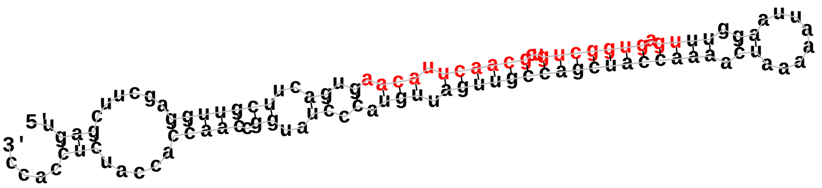

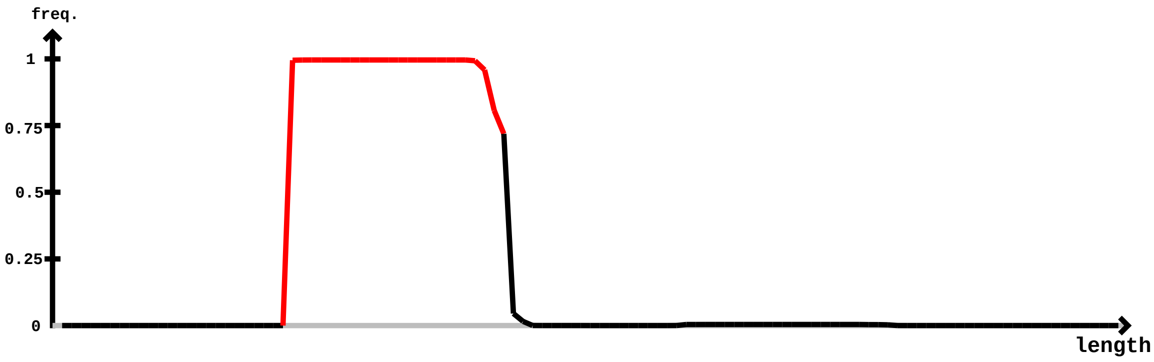


```
                          oar-miR-181a
5'- ugagcuucgagguugcuucagugaacauucaacgcugucggugaguuuggaauuaaaaaucaaaaccaucgaccguugauuguacccuauggccaaccaccaucuccacc  -3'   exp
    .(((......(((((((..((.(.(((.((((((..(((((((.((((.((........)).)))))))))))))))))).))).).))..)).))))).....)))....    reads    mm    sample
    ......................aacauucaacgcugucgg......................................................................    1        0     seq
    ......................aacauucaacgcugucggu.....................................................................    64       0     seq
    ......................aacauucaacgcugucggug....................................................................    743      0     seq
    ......................aacauucaacgcugucgguga...................................................................    3282     0     seq
    ......................aacauucaacgcugucggugag..................................................................    1887     0     seq
    ......................aacauucaacgcugucggugagu.................................................................    14534    0     seq
    ......................aacauucaacgcugucggugaguu................................................................    639      0     seq
    ......................aacauucaacgcugucggugaguuu...............................................................    330      0     seq
    ......................aacauucaacgcugucggugaguuug..............................................................    4        0     seq
    ......................aacauucaacgcugucggugaguuuggaauu.........................................................    2        0     seq
    .......................acauucaacgcugucggugag..................................................................    1        0     seq
    .......................acauucaacgcugucggugagu.................................................................    17       0     seq
    .......................acauucaacgcugucggugaguuu...............................................................    1        0     seq
    ........................cauucaacgcugucgguga...................................................................    1        0     seq
    ........................cauucaacgcugucggugagu.................................................................    2        0     seq
    ........................cauucaacgcugucggugaguuu...............................................................    1        0     seq
    ...............................................................aaccaucgaccguugauugu...........................    1        0     seq
    ...............................................................aaccaucgaccguugauuguac.........................    2        0     seq
    ...............................................................aaccaucgaccguugauuguacc........................    2        0     seq
    ................................................................accaucgaccguugauugu...........................    9        0     seq
    ................................................................accaucgaccguugauugua..........................    3        0     seq
    ................................................................accaucgaccguugauuguac.........................    14       0     seq
    ................................................................accaucgaccguugauuguacc........................    54       0     seq
    ................................................................accaucgaccguugauuguaccc.......................    2        0     seq
    ..................................................................caucgaccguugauuguacc........................    1        0     seq
```
